# Burkholderia cenocepacia Keratitis With Exogenous Endopthalmitis: A Complex Combination

**DOI:** 10.7759/cureus.95387

**Published:** 2025-10-25

**Authors:** Nur Sakinah Bahaman Shah, Abdul Hadi Rosli, Khairidzan Mohd Kamal, Muhammad Faiz Nordin

**Affiliations:** 1 Department of Ophthalmology, Kulliyyah of Medicine, International Islamic University Malaysia, Bandar Indera Mahkota, MYS

**Keywords:** burkholderia cenocepacia, burkholderia cepacia complex, endophthalmitis, keratitis, post vitrectomy

## Abstract

In this report, we present a case of *Burkholderia cenocepacia* (*B. cenocepacia*)keratitis with exogenous endophthalmitis in a post-vitrectomy eye. The patient was a 68-year-old man with a history of treated corneal laceration and endophthalmitis who presented with acute pain, redness, and lid swelling affecting the left eye following working at a palm oil plantation. There was a paracentral corneal stromal infiltrate with dense vitritis in his left eye. Cornea cultures grew *B. cenocepacia. *He received two modalities of antimicrobials, including intravitreal injections and intensive topical antibiotics. Pars plana vitrectomy was not performed due to poor corneal visibility from a previous trauma scar. His stromal infiltrates and vitreous opacity responded to therapy that was sensitive to this organism, and the keratitis healed with scarring after a month. The progression of *B. cenocepacia* keratitis to endophthalmitis can be rapid. Early identification and appropriate commencement of antibiotics can prevent detrimental sequelae, including blindness and loss of the eyeball.

## Introduction

*Burkholderia cenocepacia* (*B. cenocepacia*) is a Gram-negative bacillus bacterium that can be found commonly in soil, water, and plants [1]. It is one of the organisms under the highly virulent group known as *Burkholderia cepacia* complex (Bcc) [1]. The Bcc are aerobic, non-glucose-fermenting, free-living, motile organisms and comprise nine genomovars, namely *B. cepacia* (genomovar I), *B. multivorans* (genomovar II),* B. cenocepacia* (genomovar III), *B. stabilis* (genomovar IV), *B. vietnamiensis* (genomovar V), *B. dolosa* (genomovar VI), *B. ambifaria *(genomovar VII), *B. anthina* (genomovar VIII), and *B. pyrrocinia* (genomovar IX) [2]. *B. cenocepacia* and *B. multivorans *are the most common genomovars that mostly target immunocompromised people [3]. These organisms can cause more aggressive infections leading to an inevitable fatal outcome [1]. In the eyes, *B. cenocepacia* can result in two severe forms of ocular infections, including keratitis and endophthalmitis [4-5]. The incidence of *B.​​​​*​​​* cenocepacia *keratitis* *is limited [5]. The majority of the keratitis and endophthalmitis reported cases are caused by *B. cepacia *infection [4,6,7]*.* In this report, we discuss a case of *B. cenocepacia *keratitis with endophthalmitis in a post-vitrectomy eye.

## Case presentation

**Figure 1:**
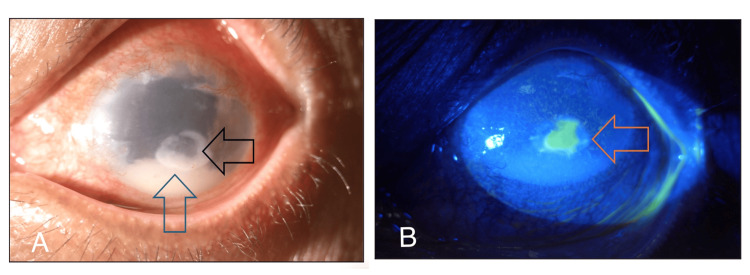
(A, B) The left anterior segment showing injected conjunctiva, paracentral immune ring (black arrow), hypopyon (blue arrow), and epithelial defect (orange arrow).

**Figure 2:**
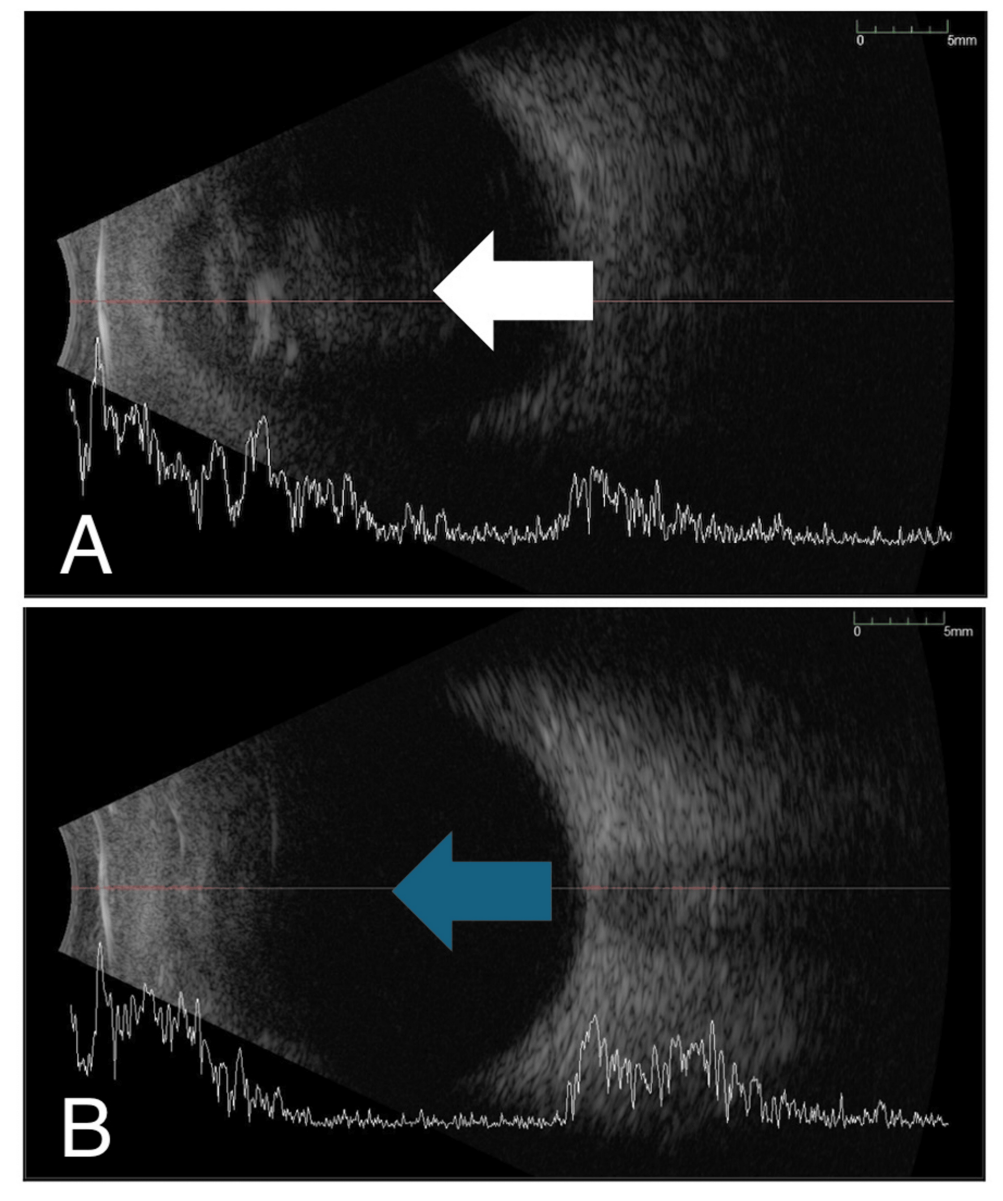
B scan showing dense vitreous loculation at initial presentation (A; white arrow) and improvement after day 4 of treatment (B; blue arrow).

A 68-year-old man with no known medical illness presented with left eye pain for six days following exposure to dust particles at a palm plantation. This was associated with a few episodes of eye rubbing, eye redness, and swelling of the lid. He denied wearing safety goggles. The left eye had a prior history of treated corneal laceration due to trauma from a sharp object and complicated cataract surgery with postoperative endophthalmitis six years ago. He underwent phacoemulsification converted to extracapsular cataract extraction with anterior vitrectomy and anterior chamber intraocular lens implantation, followed by pars plana vitrectomy and intravitreal antibiotic five days following the surgery for postoperative endophthalmitis. Prior to this presentation, his vision was 20/80. On examination, the left eye's best corrected vision was hand movement with a positive relative afferent pupillary defect. The left eyelid was minimally swollen and tender to the touch with injected conjunctiva. The cornea appeared hazy with stromal infiltrate of size 2.6 mm (V) x 3.2 mm (H) and an epithelial defect measuring 2 mm (V) x 2 mm (H). There was a 3 mm level of hypopyon with anterior chamber cells 3+ and multiple whitish opacities on the surface of the anterior chamber intraocular lens (Figure 1). The left fundus appeared hazy due to dense vitritis. B-scan ultrasound showed dense vitreous opacity with the surrounding retina being flat (Figure 2). Immediate corneal scrapings and vitreous tapping, and intravitreal injection of vancomycin (2.0 mg/0.1 ml) and ceftazidime (2.0 mg/0.1 ml) were performed. The patient was started on intensive topical medication consisting of topical gentamicin 1% and ceftazidime 5% hourly around the clock and intravenous ciprofloxacin 400 mg twice a day (BD).

**Figure 3:**
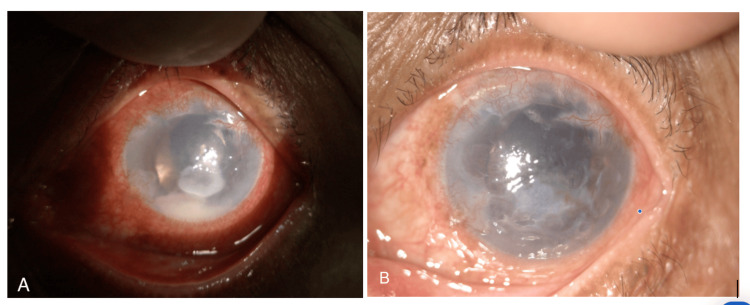
The left anterior segment showing improvement at 48 hours (A) and resolution with corneal scar at one month (B).

Corneal scraping resulted in *B. cenocepacia*, whereas the vitreous culture was negative. It was sensitive to ceftazidime, bactrim, and meropenem. Pars plana vitrectomy was not performed because of the presence of a corneal scar and limited posterior view. Therefore, conservative management was essential. After 48 hours, further improvement was noted with a well-demarcated margin of keratitis, reduced height of hypopyon, clearer cornea, and less marked vitreous opacity. He responded to medical treatment, and his medications were revised. Second intravitreal injections of vancomycin and ceftazidime were performed, and his medications were tapered weekly. The corneal ulcer and vitritis completely resolved after one month. However, his vision in the left eye remained hand movements due to a corneal scar (Figure 3).

## Discussion

Endophthalmitis is a sight-threatening condition that results from inflammation of the internal ocular cavities caused by infection. The incidence of microbial keratitis progressing to endophthalmitis is relatively uncommon [8]. Henry et al. reported that fungi were the most common responsible organism, followed by Gram-positive bacteria and Gram-negative bacteria, respectively [8]. For Gram-negative organisms, *Pseudomonas aeruginosa* (*P. aeruginosa*) was found to be the leading pathogen worldwide, causing keratitis-related endophthalmitis, which contradicts our case, indicating a rare entity [9]. Behera et al. first reported a case of keratitis caused by *B. cenocepacia* following trauma and contamination with infected soiled water from cement mix. Her patient presented with vision of hand movement and had a history of failed keratoplasty for decompensated cornea due to childhood trauma and secondary glaucoma, post-glaucoma drainage implant with pseudophakia, which requires long-term steroid use [5]. Whereas our patient's risk factors are almost similar, with previous ocular disease and trauma, previous ocular surgeries, and use of topical steroids. However, her case did not result in endophthalmitis. The absence of a natural lens-capsular barrier in our patient due to prior surgery possibly facilitated the spread from the anterior to the posterior segment, causing endophthalmitis. Additionally, the above risk factors made him more susceptible to sequential endophthalmitis, consistent with previous reported studies [5, 8-10]. 

*P. aeruginosa* and *B. cenocepacia* are two multidrug-resistant and biofilm-forming pathogens, which cause them to be extremely difficult to eradicate [11]. *P. aeruginosa* is more virulent than *B. cenocepacia*, as reported based on animal models [12]. Clinically, *B. cenocepacia* infection may mimic *P. aeruginosa* with similar signs such as moderate to severe eye pain, conjunctival injection, hypopyon, and vitritis [4, 13]. Lin et al. reported that keratitis-related endophthalmitis by *P. aeruginosa* commonly had a worse visual acuity at initial presentation, varying from hand movement or lower, as it typically presented with severe suppurative infection [14]. Generally, the final visual outcome in *P. aeruginosa* endophthalmitis is poor, with the final visual outcome being light perception or no light perception and a higher rate of evisceration (42%) [14]. Whereas, for *B. cenocepacia* infection, it demonstrated variable presentation and varied final outcomes from case to case. Based on a literature review by Beca et al. for Bcc endophthalmitis, it was reported that 25% had final vision of 20/40 or better, 32% had between 20/40 and 20/200, and seven patients ended with phthisis or enucleation [4]. Despite a cleared infection in our case, the patient's final vision remained hand movement due to a corneal scar.

Considering we are living in a tropical country and the patient had been exposed to a palm oil plantation, fungal infection can also be one of the differentials. However, the treatment approach was initially started with standard recommendations of empirical broad-spectrum intravitreal and topical antibiotics covering both Gram-positive and Gram-negative bacteria until the culture results were out. In our case, *B. cenocepacia* isolated was susceptible to ceftazidime, bactrim, and meropenem, consistent with previous findings [4, 15]. Ceftazidime was the most commonly tested and most commonly sensitive antibiotic from various published reports [4]. Despite being sensitive to ceftazidime, Deb et al. reported that five of their patients who were initially treated with vitreous tap and injection of vancomycin and ceftazidime ultimately required additional pars plana vitrectomy due to inadequate initial response. Three of them had subsequent recurrences and were treated with intravitreal imipenem, intravenous meropenem for a week, and topical fortified ceftazidime for eight to 12 weeks [16]. Unlike our patient, pars plana vitrectomy was deferred due to poor corneal visibility and his good response to the initial treatments. Antimicrobial testing done by Lama et al. found that the *B. cepacia* variant possesses several antimicrobial resistances, including to fluoroquinolones, cephalosporins, carbapenems, monobactams, aminoglycosides, and sulfonamides [17]. Despite that, Behera et al. proved that topical monotherapy with antibiotics (moxifloxacin 0.5%) can also be used, and the ulcer healed in one month [5]. 

## Conclusions

In conclusion, *B. cenocepacia* remains a significant pathogen in ocular infections. The usage of personal protective eyewear at work can be used as a preventative measure to reduce eye injury and exposure to this pathogen. Keratitis-related endophthalmitis requires multimodal therapeutic strategies involving intravitreal antibiotics, topical agents, and systemic antibiotics. Certain cases may require additional pars plana vitrectomy. Although the final outcome is guarded, it can vary from case to case. Therefore, early identification, appropriate commencement of antimicrobial therapy, and judicious use of steroids at an early stage are crucial in managing this rare entity. 
